# The P2X7 Receptor in Osteoarthritis

**DOI:** 10.3389/fcell.2021.628330

**Published:** 2021-02-11

**Authors:** Zihao Li, Ziyu Huang, Lunhao Bai

**Affiliations:** ^1^Department of Orthopedic Surgery, Shengjing Hospital of China Medical University, Shenyang, China; ^2^Foreign Languages College, Shanghai Normal University, Shanghai, China

**Keywords:** osteoarthritis, P2X7 receptor, inflammation, apoptosis, pyroptosis, autophagy

## Abstract

Osteoarthritis (OA) is the most common joint disease. With the increasing aging population, the associated socio-economic costs are also increasing. Analgesia and surgery are the primary treatment options in late-stage OA, with drug treatment only possible in early prevention to improve patients’ quality of life. The most important structural component of the joint is cartilage, consisting solely of chondrocytes. Instability in chondrocyte balance results in phenotypic changes and cell death. Therefore, cartilage degradation is a direct consequence of chondrocyte imbalance, resulting in the degradation of the extracellular matrix and the release of pro-inflammatory factors. These factors affect the occurrence and development of OA. The P2X7 receptor (P2X7R) belongs to the purinergic receptor family and is a non-selective cation channel gated by adenosine triphosphate. It mediates Na^+^, Ca^2+^ influx, and K^+^ efflux, participates in several inflammatory reactions, and plays an important role in the different mechanisms of cell death. However, the relationship between P2X7R-mediated cell death and the progression of OA requires investigation. In this review, we correlate potential links between P2X7R, cartilage degradation, and inflammatory factor release in OA. We specifically focus on inflammation, apoptosis, pyroptosis, and autophagy. Lastly, we discuss the therapeutic potential of P2X7R as a potential drug target for OA.

## Introduction

Osteoarthritis (OA), as an age-related degenerative joint disease, presents with physical pain and disability in patients. Finding effective therapies for OA remains a pertinent health problem (Carr et al., 2012). The occurrence and development of OA can be attributed to multifaceted factors, such as age, obesity, exercise, and trauma (Uthman et al., 2013). From a clinical perspective, its features include articular cartilage loss, synovitis, subchondral bone sclerosis, and osteophyte formation. From a cellular perspective, it is attributed to morphological, biochemical, and biomechanical changes affecting the extracellular matrix (ECM).

The most important structural part of the joint is the cartilage, and its condition directly affects the occurrence and development of OA. Cartilage consists of structural proteins, such as collagen (primarily type II collagen), non-collagen proteins, proteoglycan, elastin, and aminoglycan, which form a stable network structure providing elasticity and compression resistance to joints. In OA tissues, this network structure loses its integrity with a resulting loss of tensile strength (Speziali et al., 2015). The composition and integrity of the cartilage matrix can be maintained by chondrocytes—which account for a small proportion of the total cartilage volume—to provide mechanical support and joint lubrication (Archer and Francis-West, 2003). In response to certain chemical and mechanical factors, chondrocytes produce and release inflammatory factors (IL-1β, IL-6, and TNF-α) and secrete matrix-degrading enzymes [metalloproteinases (MMPs)] and proteoglycan-degrading enzymes (ADAMTS), to regulate the shape and structure of cartilage. Unfortunately, these inflammatory factors and enzymes are the primary contributors to cartilage degradation. Therefore, the occurrence and development of OA can be ascribed to the state and the catabolism balance of chondrocytes and cartilage. In OA, the changes in the chondrocytes can be categorized into three groups, namely: (i) cell proliferation (early stage)/cell death (late stage); (ii) anabolic and catabolic balance disorder (production of matrix-degrading enzymes); and (iii) phenotypic change (Sandell and Aigner, 2001). The influence of the cell phenotypic changes in the development of OA is particularly critical. In this article, we focus on chondrocyte inflammation, apoptosis, pyroptosis, and autophagy, and analyze the correlation between these phenotypes and cartilage degradation in OA.

The P2X7 purinergic receptor (P2X7R) is a trimeric adenosine triphosphate (ATP)-gated cation channel, which is expressed in several eukaryotic cells, such as immune cells and bone cells. As a key inflammatory switch, the activation of P2X7R mediates several downstream reactions, including the release of inflammatory factors, cell proliferation, death, and phenotypic changes (North, 2002). Owing to the important role of P2X7R in various immune, inflammatory, musculoskeletal, and nervous system diseases, it could be a potential drug treatment target for OA. In this article, we review the association between P2X7R and OA. We explain the structure and function and the inhibitory effect of P2X7R and emphasize the correlation and intersection between P2X7R, inflammation, and OA. From the perspective of apoptosis, pyroptosis, and autophagy, we discuss the possible association between P2X7R, cartilage degradation, and inflammatory factor release in OA. Further, we summarize the possible treatment methods, including the use of P2X7R as a drug target, and highlight the potential future mechanistic research.

## Elucidation of OA and Its Treatment

### Focus on Preventing Inflammation

Most studies on OA focus on the synovium-mediated development of inflammation. Synoviocytes identify the foreign fragments that fall into the joint cavity as byproducts of cartilage degradation. This results in synovial angiogenesis, an increase in the release of auto-inflammatory factors, and the stimulation of chondrocytes to synthesize and secrete MMPs. Through this mechanism, synovitis can promote cartilage degradation and aggravate OA (van Lent et al., 2004). Synovial fluid factors are increased in the damaged cartilage of OA patients (Kim et al., 2006), including toll-like receptor (TLR)-2 and TLR-4 ligands, such as alarm proteins [S100 protein and high mobility group protein B1 (HMGB1)], low molecular weight hyaluronic acid, tenascin C, and fibronectin (Scanzello et al., 2008; García-Arnandis et al., 2010; van Lent et al., 2012). These mediators, together with low-grade inflammation present in the compromised joints, induce inflammation of the synoviocytes and affect the catabolism balance of the chondrocytes (Wang et al., 2011). Therefore, the innate immunity may be a key factor driving the development of OA. As for mild systemic inflammation, plasma and peripheral white blood cells can reflect the level of inflammation in the joint tissues. Adipokines secreted from visceral fat, such as leptin, resistin, and adiponectin also play an important role (Gomez et al., 2011), having both pro- or anti-inflammatory effects on OA development (Distel et al., 2009). The release of inflammatory factors into the blood can cause diseases in other parts as well, such as the inflammation of the nervous system and Alzheimer’s disease (Kyrkanides et al., 2011).

### Clinical Treatment Options

Although our understanding of the mechanism underlining OA has improved, limited progress has been made with respect to its treatment. Currently, analgesia and joint replacement are primarily used to treat end-stage OA (Bijlsma et al., 2011; Carr et al., 2012; Pivec et al., 2012) which neglects the problem of early disease incidence. Fortunately, the continuous advancement in our understanding of OA pathogenesis and the improvement in detection methods have shifted the focus toward the prevention and treatment of early OA. Lifestyle adjustments, such as weight loss and exercise for obese individuals, can enhance muscle strength and joint stability, improve cardiovascular function, and reduce the risk of OA (Felson et al., 1992; Gudbergsen et al., 2012; Uthman et al., 2013).

Drug treatment often goes hand-in-hand with prevention, with commonly used drugs in clinical practice, such as paracetamol (acetaminophen) and non-steroidal anti-inflammatory drugs (NSAIDs) also being able to effectively relieve pain symptoms (Palmer et al., 2013). Furthermore, the anti-inflammatory and anti-catabolism properties of chondroitin and glucosamine have been proven in clinical trials to alleviate the occurrence and development of OA (Henrotin and Lambert, 2013). Hyaluronic acid, a glycosaminoglycan, can also act as a lubricant in the synovial fluid. In patients with OA, the concentration of hyaluronic acid in the joint cavity is low, and the friction experienced by the joint surfaces during limb movement increases pain. It must be noted, however, the clinical efficacy and safety of injecting hyaluronic acid into the joint cavity remains controversial (Rutjes et al., 2012). Another lubricating element, lubricin, has been shown to work synergistically with hyaluronic acid with limited effects (Schmidt et al., 2007). Several targeted drugs have also been developed to treat OA, such as MMP inhibitors (doxycycline) (da Costa et al., 2012), osteoclast inhibitors (bisphosphonates and strontium ranelate) (Henrotin et al., 2001), IL-1R inhibitors (anakinra) (Chevalier et al., 2009), immunoglobulins (AMG 108) (Cohen et al., 2011), TNF-α inhibitors (adalimumab) (Verbruggen et al., 2012), cartilage repair factors [recombinant osteogenic protein-1 (Nishida et al., 2004) and kartogenin (Johnson et al., 2012)]. The application of these drugs still needs improvement as there is still a gap between the desired therapeutic effect and the clinical outcomes. This implies that more effective and targeted drugs are required in the prevention and treatment of OA. Therefore, an in-depth exploration of the particular etiology and pathogenesis of OA is critical.

## The P2X7 Receptor

### P2X7R Structure and Function

Purine receptors can be divided into two categories: adenosine (ADO) activated P1 receptors and purine and pyrimidine nucleotides (ATP and ADP) activated P2 receptors. P2 receptors can further be divided into P2X ion channel receptors and P2Y metabotropic receptors. There are seven different members of the P2X family (P2X1-7), of which the P2X7 receptor (P2X7R, encoded by the *P2RX7* gene) is most closely related to inflammation and immunity, and belongs to the trimeric ligand-gated cation channel. Compared with other P2X receptors, P2X7R requires a higher concentration of ATP for activation and has a higher affinity for the selective agonist BzATP, with 10–30 times more potency than ATP (North, 2002). In addition, natural splice variants (P2X7A-J) and (P2X7a, P2X7k, P2X713b, and P2X713c) were found in human and rodent tissues, respectively, with P2X7R sharing 77–85% sequence identity. Therefore, several experiments have used rodent models to study the function of P2X7R.

Structurally, P2X7R contains relatively short intracellular amino- (N) and long carboxyl- (C) termini, and two hydrophobic transmembrane fragments separated by glycosylated extracellular ATP binding domains (transmembrane domains); its topology is similar to that of other ionic P2X receptors (Khakh and North, 2006; Karasawa and Kawate, 2016). The functional channel toward the plasma membrane is composed of stable trimers (Nicke, 2008; Jiang et al., 2013). Moderate activation occurs when the receptor is bound to ATP. The gated state of P2X7R is opened, mediating non-inactivated Na^+^ and Ca^2+^ influx and K^+^ efflux, resulting in rapid depolarization (Surprenant et al., 1996). When the activation time is prolonged, P2X7R can induce the formation of membrane pores, allowing molecules up to hundreds of Da to pass (Pelegrin and Surprenant, 2006).

### P2X7R Activation and Regulation

The transcription and expression of P2X7R can be regulated by microRNAs [e.g., miR-373 (Zhang et al., 2018)], long-coding RNAs (e.g., lncRNA NONRATT021972), and transcription factors [e.g., specific protein 1 (Sp1)], and P2X7R will also undergo post-translational modifications, including N-linked glycosylation, palmitoylation, and ADP-ribosylation (Sluyter, 2017). The most important mediator for the activation of receptors is ATP and its role in inflammation and immunity has been proven. Cell death in inflammatory tissues releases large amounts of ATP, increasing the extracellular ATP concentration to hundreds of μM, which is enough to activate P2X7R. In contrast, the extracellular ATP concentration in healthy tissues is very low (Pellegatti et al., 2008; Wilhelm et al., 2010; Barbera-Cremades et al., 2012). In addition to passive release, ATP can also cross incomplete cell membranes through specific membrane protein channels, or can be stored in cytoplasmic vesicles and secreted outside the cell (Burnstock, 2006). Other mechanisms, such as the one mediated by the gap junction protein pannexin-1, control the release of ATP from living or apoptotic cells. Under certain conditions, such as hypoxia, increased extracellular K^+^ concentration, and mechanical stress stimulation, pannexin-1 has high ATP permeability (Wang and Dahl, 2018). In undamaged tissues, a small amount of ATP in the extracellular environment will be rapidly degraded by enzymes (Plesner, 1995). Therefore, to activate P2X7R, the ATP release channel and the receptor should be in close proximity, which explains the closely related protein functions and structures of pannexin-1 and P2X7R (Bao et al., 2004; Pelegrin and Surprenant, 2006; Locovei et al., 2007).

The P2X7 receptor activation mediates numerous signaling pathways and cellular responses, such as the activation of the NLRP3 inflammasome via K^+^ efflux, which induces IL-1β release; the formation of mitochondrial reactive oxygen species (mtROS) via ATP signaling, which can also regulate the activity of P2X7 ion channels; the regulation of caspase, cathepsin, and MMP release; and the regulation of the pro-inflammatory mediator prostaglandin E2 (PGE-2). PGE-2 is an important downstream inflammatory pathway of P2X7R, which may make P2X7R a potential anti-inflammatory target to replace cyclo-oxygenase that regulates the activation of transcription factors, such as NF-κB p65, HIF-1α, and PI3K-AKT; glutamate efflux; endocytosis; cell proliferation; and cell death. Briefly, P2X7R regulates ion flow, protease activation, and various secretory responses, which constitute the most common signaling pathways in inflammation (Bartlett et al., 2014). Therefore, P2X7R is also known as an inflammatory switch.

## P2X7R and Osteoarthritis

The importance of P2X7R in inflammation has recently attracted attention in the field of bone-related diseases. It has critical influence on OA, synovitis, and rheumatoid arthritis. This has opened a way for P2X7R inhibitors (as a specific target-directed approach) to serve as potential new therapeutics for these diseases.

### P2X7R as an Inflammatory Switch

When OA occurs, the most significant cellular response is inflammation, which can be triggered by external and internal mechanisms. External factors include mechanical stress (compression, stretching, hydrostatic pressure, and shear stress), while the mechanoreceptors (ion channels and integrins) present on the surface of joint cells convert abnormal mechanical stress into activated intracellular signals (Guilak, 2011). Accordingly, the activation of the NF-κB and mitogen-activated protein kinase (MAPK) signaling pathways is commonly observed (Signaling, 2004).

#### Exercise as an Anti-inflammatory Mechanism

In daily life, physical exercise causes mechanical stress on joints that can be beneficial. Studies have shown that regular exercise can alleviate low-intensity inflammatory conditions, as are observed in OA, cancer, and cardiovascular disease, and reduce the risks associated with a high-fat diet. P2X7R is one of the factors that determine the level of IL-1β in the plasma after exercise. Furthermore, P2X7R, NF-κB, NLRP3, and caspase-1 levels have been shown to progressively increase between sedentary individuals, trained athletes, and endurance athletes (Comassi et al., 2017). Post-exercise, NLRP3 and caspase-1 levels increased in sedentary individuals (pro-inflammatory), while they decreased in endurance athletes (anti-inflammatory). Regardless of the degree of fitness, acute exercise increased P2X7R expression and function. In another study, exercise reduced the expression levels of P2X7R, NLRP3, caspase-1, and IL-1β in plasma caused by a high-fat diet in Sprague Dawley rats, inhibiting inflammation and apoptosis, enhancing autophagy, and reducing myocardial damage (Chen et al., 2019). It is thus evident that P2X7R plays an important role during exercise to relieve inflammation and other risk factors.

#### Cytokine Secretion During Inflammation

The P2X7 receptor regulates the intensity and duration of several inflammatory reactions (Ferrari et al., 2006; Khakh and North, 2006; Dubyak, 2012). In macrophages, monocytes, and microglia, P2X7R mediates Ca^2+^ influx and K^+^ efflux inducing the activation and release of cytokines (Kahlenberg and Dubyak, 2004; Ferrari et al., 2006). This initiates inflammasome assembly, while caspase-1 pro-IL-1β cleavage releases a large amount of mature IL-1β (MacKenzie et al., 2001; Pelegrin et al., 2008; Dubyak, 2012). IL-1β can induce reactive oxygen species (ROS) and the expression of protein-degrading enzymes, leading to the loss of type II collagen and proteoglycans, thereby destroying cartilage structure and affecting joint function and stability (Sitia and Rubartelli, 2020). IL-1β is an atypical cytokine lacking secreted fragments which does not follow the standard endoplasmic reticulum-Golgi pathway for extracellular release (Dinarello, 2002). Instead, it functions via an ATP-dependent P2X7R-inflammasome pathway by TLR stimulation-induced accumulation of pro-IL-1β in the cytoplasm followed by its subsequent release (Perregaux et al., 2000; Ferrari et al., 2006). Other mechanisms of cytokine release occur through passive release after cell death, and secretion by modified lysosomes, exosomes, or plasma membrane-derived microvesicles (MacKenzie et al., 2001; Lopez-Castejon and Brough, 2011; Piccioli and Rubartelli, 2013). In all these instances, P2X7R is the main driver (Bianco et al., 2005; Pizzirani et al., 2007; Qu et al., 2007), further emphasizing its key role in the release of biologically active IL-1β.

#### Oxidative Stress in Inflammation

Among the internal factors contributing to the development of OA, ROS play a pivotal role. ROS comprise molecules containing free radicals, including oxygen free radicals (OH^–^), hypochlorite ions (OCl^–^), superoxide anions (O_2_^–^), nitric oxide (NO), and hydrogen peroxide (H_2_O_2_). ROS is mainly produced through the following pathways: mitochondrial (through oxidative phosphorylation) and non-mitochondrial membrane-bound nicotinamide adenine dinucleotide phosphate (NADPH) oxidase and xanthine oxidase (XO) pathways (Turrens, 2003). When the catabolic cell balance is disturbed, the accumulation of ROS leads to an increase in the production of inflammatory mediators, and an ineffective elimination of the oxidized proteins. This ultimately triggers oxidative damage that exacerbates inflammation (Licastro et al., 2005). Oxidative stress can promote cell senescence, especially affecting chondrocytes (Loeser, 2011). Chondrocytes have a low ability to divide and proliferate with high ability to synthesize and secrete, a circumstance called the senescence-associated secretory phenotype (SASP) (Coppe et al., 2010). ROS normally occurs at low levels in chondrocytes but can still regulate gene expression, affect ECM synthesis and catabolism balance, drive the production of cytokines, such as IL-1β (Forsyth et al., 2005), and induce cell apoptosis. The exacerbated oxidative stress in chondrocytes inhibits the PI3K/Akt pathway; activates the NF-κB pathway to promote the transcription of MMPs; activates the extracellular signal-regulated kinase (ERK)/MAPK pathway to reduce the expression of type II collagen, proteoglycans, and Sox-9; reduces ECM synthesis (Yin et al., 2009; Yu and Kim, 2015); and acts as a signal transduction intermediate for IL-1β and TNF-α in the c-Jun N-terminal kinase (JNK) pathway activation (Lo et al., 1996). Therefore, ROS plays an important role in the intracellular signal transduction mechanisms and is closely related to cartilage homeostasis (Rathakrishnan et al., 1992; Henrotin et al., 1993; Henrotin et al., 2003).

#### Significance of P2X7R in Inflammation

Summarily, the ion flow mediated by P2X7R activation is closely related to the homeostasis of the intracellular environment. Mitochondrial dysfunction caused by Ca^2+^ overload can induce the production of ROS. Activation of inflammasomes triggered by K^+^ efflux can induce the production of IL-1β and the activation of inflammation-related pathways. This indicates the important role of P2X7R in inflammation and its potential influence on the occurrence and development of OA. In terms of preventive treatment and cytokine treatments were not found to significantly improve the symptoms of OA or relieve the deterioration of bone structure. The results of pilot and controlled studies using anti-IL-1 and anti-TNF molecules lack credibility (Chevalier et al., 2009; Verbruggen et al., 2012). In this context, P2X7R-targeted therapy could present as a new direction for the prevention and treatment of OA.

### P2X7R Induces Apoptosis in OA

When a cell undergoes apoptosis, the chromatin condenses around the nucleus, the cell membrane shrinks and bubbles, and apoptotic bodies with intact membranes form around organelles (Kerr et al., 1972). Upon recognition by the immune system, inflammation is induced. This can be prevented, however, by the phagocytic engulfment of these vesicles (Kurosaka et al., 2003). Apoptosis is a programmed cell death, which differs from passive necrosis (or cell membrane disintegration) caused by pyroptosis. At the molecular level, initiation, execution, degradation, and clearance are the established sequential steps of apoptosis.

#### Apoptotic Pathways

Apoptosis can be induced via extrinsic mediation by death receptors and via intrinsic mediation by mitochondria (Elmore, 2007). The extrinsic pathways include damage or pathogen-related molecular patterns (DAMPs or PAMPs) and cytokines that activate the TNF superfamily (e.g., death receptors). The death receptor Fas and its ligand FasL combine to drive the assembly of the death-inducing signaling complex (DISC). Thereafter, the recruitment and activation of caspase-8 are mediated by Fas-related proteins and death domain (FADD) adaptor molecules, which leads to caspase-3 activation, and finally apoptosis (Fuentes-Prior and Salvesen, 2004). P2X7R is closely related to apoptosis. IL-1β, mediated by P2X7R, can further induce the production of TNF-α, both having pro-apoptotic effects (Lee et al., 2016). The membrane pores formed by P2X7R (Donnelly-Roberts et al., 2004), together with its mediated K^+^ efflux (Delarasse et al., 2009; Aguirre et al., 2013), can induce the activation of caspase-8 and subsequently cleave caspase-3, the key executor of apoptosis.

Intrinsic pathways include those mediated by DNA damage, cytoplasmic Ca^2+^ overload, oxidative or endoplasmic reticulum stress (ERS), decreased cytokine levels, and the response to intracellular damage (Vanden Berghe et al., 2015). Bcl-2 family members respond by changing the permeability of the mitochondrial outer membrane (Kroemer and Reed, 2000). The activation of its priming members (e.g., Bid) not only inhibit survival supervisory members (e.g., Bcl-2), but also oligomerize pro-apoptotic members (e.g., Bax), thereby destroying the mitochondrial outer membrane. A large number of proteins are released into the cytoplasm, such as cytochrome c (Cyt c) (Qiu et al., 2000), activating a key component of apoptotic bodies, namely the apoptotic protease activator factor-1 (APAF-1) (Green, 2003). Cyt c combines with oligomeric APAF-1 to form a multimeric structure, recruiting and activating caspase-9 in the mitochondrial pathway (Bao and Shi, 2007). Caspase-9 directly activates downstream caspase-3, 6, and 7, ultimately leading to substrate cleavage and apoptosis. In addition to the inflammasome assembly caused by the intracellular K^+^ consumption, K^+^ efflux can also induce apoptosis by promoting APAF-1 (Bortner et al., 1997; Karki et al., 2007). Furthermore, Ca^2+^ influx can lead to mitochondrial dysfunction and caspase-3 activation. As P2X7R plays a crucial role ion transport, P2X7R inhibitors have shown promise in preventing cell death dependent on these mechanisms (Nishida et al., 2012).

#### Oxidative Stress in Apoptosis

Reactive oxygen species (e.g., H_2_O_2_) damages mitochondria as part of the apoptotic intrinsic pathway, destroying its DNA integrity and repair ability (Grishko et al., 2009). This can induce chondrocyte apoptosis through PI3K/Akt, p38 MAPK, and JNK signaling pathways (Yu and Kim, 2014; Li and Dong, 2016; Rao et al., 2017). As mentioned earlier, P2X7R can induce the generation of ROS, and the activation of P2X7 will increase the phosphorylation of tyrosine protein, activating MAPK (Panenka et al., 2001; Papp et al., 2007), including ERK, JNK, and p38 MAPK. ERK is essential for cell survival, while JNK and p38 MAPK can be activated under stress stimulation (Wada and Penninger, 2004; Yang et al., 2012). JNK phosphorylates Bcl-2 to reduce its anti-apoptotic activity (Cui et al., 2007), and p38 MAPK reduces the expression of Bcl-2 and induces the production of caspase-3, leading to apoptosis (Nishida et al., 2012). The NF-κB signaling pathway closely relates to P2X7R involvement in chondrocyte apoptosis, since it can affect the expression of apoptosis-related proteins (Bcl-2, Bax, Cyt c, and caspase-3) (Pan et al., 2018).

#### Chondrocyte Apoptosis

Unregulated apoptosis can lead to pathological changes, such as tumors and inflammation. Apoptosis is both the initiator (Hashimoto et al., 1998) and feedback in the development of OA (Blanco et al., 1998; Heraud et al., 2000). Specifically, chondrocyte apoptosis is considered a prerequisite for the development of OA. Compared with the normal cartilage, the number of apoptotic chondrocytes is increased in the cartilage of OA patients (Hashimoto et al., 1998), while that of normal chondrocytes is decreased (Aigner et al., 2006), and a dysregulation of apoptosis-related genes occurs (Bobinac et al., 2003). In addition, animal studies showed that low levels of mechanical stress stimulation can induce chondrocyte apoptosis, with high levels of mechanical stress stimulation leading to the degradation of the cartilage matrix (Loening et al., 2000). This indicates that chondrocyte apoptosis occurs first, followed by tissue damage, reflecting early features of OA. Therefore, chondrocyte apoptosis also represents the feedback of the outcome of OA (Zamli et al., 2013). In transgenic mice with type II collagen gene knockout, chondrocytes undergo increased apoptosis, have lower survival, and no longer interact with the surrounding matrix, and there is presentation of severe cartilage degradation, compared to the wild-type mice (Zemmyo et al., 2003). Several studies have confirmed the positive relationship between the severity of cartilage degradation and the rate of chondrocyte apoptosis (Blanco et al., 1998; Hashimoto et al., 1998; Thomas et al., 2007). Some studies used collagenase to destroy the matrix, increasing cell permeability to expose the chondrocytes to apoptosis inducers (NO and cytokines) secreted by either synoviocytes or other chondrocytes, ultimately leading to cell apoptosis (Zamli and Sharif, 2011). Taken together, although the sequence of cartilage degradation and chondrocyte death is still controversial, they are undeniably closely related in OA (Zamli and Sharif, 2011).

### Interplay With Pyroptosis

Apoptosis does not always occur in isolation and often accompanies other phenotypes. In the early stage of OA, autophagy plays a protective role to prevent cell death on the surface and middle layers of cartilage (Almonte-Becerril et al., 2010; Carames et al., 2010); while in the late stage of OA, autophagy induces apoptosis and accelerates cell death (Almonte-Becerril et al., 2010). In the cartilage of patients with OA, the apoptotic bodies that have not been engulfed in time, together with increasing cartilage calcification, aggravate OA through secondary pyroptosis (Roach et al., 2004). The cell death caused by apoptosis and pyroptosis have also been confirmed in a model of OA induced by mechanical stress (Chen et al., 2001; Stolberg-Stolberg et al., 2013). *In vivo* and *in vitro* experiments showed that the application of mechanical stress to the chondrocytes of human cartilage explants (D’Lima et al., 2001), end plates (Kong et al., 2013), or growth plates (Sun et al., 2017) can lead to the loss of type II collagen, proteoglycans, and glycosaminoglycans, accompanied by cell death. The protective role of antioxidants must briefly be mentioned as they can reduce cell death caused by shear stress (Martin and Buckwalter, 2006) and abnormal cyclic loading (Beecher et al., 2007).

#### Significance of P2X7R in Apoptosis

In summary, P2X7R-mediated ion flow, oxidative stress, and related signaling pathways are closely related to apoptosis. Apoptosis is complemented by OA under the action of autophagy and pyroptosis. Thus, P2X7R plays an important role in the interaction between apoptosis and OA. It participates in cartilage degradation and inflammatory factor release, aiding the progression to OA.

### P2X7R Drives Pyroptosis in OA

Apoptosis and pyroptosis can occur independently, sequentially, and simultaneously (Zeiss, 2003). Both have common triggers and biochemical networks. The intensity and duration of different stimuli, the amount of ATP that can be consumed in the cell, and the potency of caspases can convert the ongoing process of apoptosis into pyroptosis (Leist et al., 1997; Denecker et al., 2001; Zeiss, 2003). The process of accidental and passive cell death caused by harmful stimuli (ultraviolet radiation, heat, hypoxia, and cytotoxic drugs), accompanied by cytoplasmic granulation, mitochondrial swelling, cell swelling, rupture of the plasma membrane, and uncontrolled release of intracellular substances (including pro-inflammatory cytokines, DAMPs, and lysosomal enzymes), causes an inflammatory response called pyroptosis (Festjens et al., 2006; Kaczmarek et al., 2013).

#### NLRP3 as the Initiator of Pyroptosis

NLRP3, as one of the components of the inflammasome (the core component of pyroptosis) exists in the cytoplasm and can recognize PAMPs (such as pore-forming toxins and microbial cell wall components) or DAMPs (such as ATP derived from endogenous stress and uric acid crystals) (McGilligan et al., 2013; Guo et al., 2015; Sharma and Kanneganti, 2016). NLRP3 activates caspase-1, promoting the secretion of IL-1β and IL-18 to exacerbate inflammation (Shao et al., 2015), resulting in rapid cell death (Latz et al., 2013). There are two main ways to activate inflammasomes. The first is through the recognition of PAMPs or DAMPs by TLRs to activate the NF-κB signaling pathway, increasing the synthesis of NLRP3, pro-IL-1β, and pro-IL-18; the second is by initiating oligomerization, leading to the assembly of inflammasomes (Shao et al., 2015). Both kinase activity and autophagy can regulate the activity of the NLRP3 inflammasome (Yang et al., 2017). Autophagy relieves pyroptosis by degrading pro-IL-1β, inflammasome components, and damaged mitochondria (Zhou et al., 2011; Shi et al., 2012).

As a key activator of inflammasomes, P2X7R participates in both the classical pathway (e.g., NLRP3) and non-classical pathway (e.g., caspase-11). The latter induces cell death similar to pyroptosis (Vigano and Mortellaro, 2013; de Gassart and Martinon, 2015). The C-terminal part of pannexin-1 can be cleaved by caspase-11, leading to ATP release and K^+^ efflux (Yang et al., 2015). Hypoxia-induced caspase-11 expression and activation (Kim et al., 2003) are involved in ERS-dependent cell death (Fradejas et al., 2010). P2X7R and NLRP3 can also interact physically. Immunoprecipitation and confocal microscopy studies have found that changes in the local ionic microenvironment in cells caused by P2X7R or by a membrane pore opening can result in inflammasome assembly (Franceschini et al., 2015).

Under normal circumstances, inflammasomes activate the innate immune system, producing IL-1β, IL-18, and other pro-inflammatory cytokines to protect cells from infection and reduce damage (Man and Kanneganti, 2015). However, excessive activation of inflammasomes overproduce cytokines, causing inflammation and metabolic disorders (Strowig et al., 2012). NLRP3 is an established potential target for diseases, such as atherosclerosis, rheumatoid arthritis, and gout. NLRP3 is also activated in the synovial tissue contributing to cartilage degradation (Bougault et al., 2012). In patients with OA, high NLRP3 expression in tissue has been linked to high XO levels (an enzyme producing ROS and uric acid), further confirming the association between OA and NLRP3 inflammasomes (Clavijo-Cornejo et al., 2016).

As stated before, cartilage degradation in OA can result from disrupted anabolic and catabolic chondrocyte balance (Bijlsma et al., 2011). Once initiated, the primary cartilage-degrading enzymes, IL-1β and TNF-α, are produced by the NLRP3 inflammasome (Mueller and Tuan, 2011; Haseeb and Haqqi, 2013). IL-1β both induces cell apoptosis (Haseeb and Haqqi, 2013) and stimulates the secretion of other cartilage-degrading enzymes, such as MMP3/13 and ADAMTS-4/5 (Mueller and Tuan, 2011), leading to the degradation of the type II collagen and the proteoglycans of the ECM (Haseeb and Haqqi, 2013). The released collagen and proteoglycan particles stimulate the production of IL-18 (Olee et al., 1999; Man and Mologhianu, 2014), which inhibits proteoglycan synthesis and chondrocyte proliferation, promotes prostaglandin production, and further induces apoptosis (Jin et al., 2011).

#### Significance of P2X7R in Pyroptosis

Targeting the components of inflammasomes and upstream and downstream pathways, such as K^+^ efflux, ROS, mitochondrial-, and lysosome dysfunction, is an important approach in the treatment of OA (Di Virgilio, 2013; He et al., 2016). The small molecule inhibitor MCC950 inhibits the oligomerization of inflammasomes and IL-1β release (Coll et al., 2015). Drugs that target IL-1R, such as rilonacept, anakinra, and canakinumab, can also reduce cartilage destruction (Dinarello et al., 2012). As an important driving force of the inflammasome pathway, P2X7R has potential as a drug target treatment to alleviate cartilage damage caused by pyroptosis.

### P2X7R Regulates Autophagy in OA

Cells degrade excess or damaged lipids, proteins, and organelles, to maintain cell viability through autophagy. Autophagy also plays a role in maintaining mitochondrial function (Lo Verso et al., 2014), as the downregulation of autophagy will induce the accumulation of damaged mitochondria, increase the levels of ROS, and lead to tissue degeneration (Mizushima and Komatsu, 2011). Autophagic flux can be divided into five stages: (i) induction, (ii) nucleation, (iii) extension, (iv) maturation, and (v) lysis. A complex is formed in each of the first three stages. In the induction phase, the Atg1 complex (including Atg1/Ulk1, Atg13, and Atg17/FIP200) and the activity of mammalian target of rapamycin complex 1 (mTORC1) is inhibited, Atg13 phosphorylation level is reduced, and the complex is formed (Ganley et al., 2009). The Vps34 (PI3K)-Atg6 (Beclin-1) complex acts on the nucleation of the membrane vesicles and mediates the formation of the pre-autophagosome structure. The extension of autophagosomes mainly depends on two ubiquitin-like systems: the binding process of Atg12-Atg5 and the modification process of LC3. LC3-II is a multi-signal transduction regulatory protein that is located on the autophagy vesicle membrane and is often used as a marker for autophagy formation.

#### Chondrocyte Autophagy

Cartilage lacks blood vessels to supply oxygen, thus cells exist in a somewhat hypoxic environment. Hypoxia promotes autophagy (e.g., through an increase in *Ulk1* and *Atg5* mRNA levels), maintains the chondrocyte phenotype (e.g., increased *Sox9* and *type II collagen* mRNA levels, decreased *MMP13* and *ADAMTS-5* mRNA levels), and further induces hypoxia factors (HIFs) (Coimbra et al., 2004), which play important roles as key regulators of autophagy in chondrocytes. HIF-1 is a heterodimer composed of two different subunits, HIF-1α and HIF-1β. Under hypoxic conditions, HIF-1α degradation is prevented and transferred to the nucleus, where it combines with β subunits to form an active HIF-1 transcription factor. HIF-1α is essential for cell survival, and its knockout induces mass chondrocyte death in the cartilage growth plate (Schipani et al., 2001). Bcl-2 plays a role in HIF-1α-mediated autophagy (Zhang F. et al., 2015), while HIF-1α modulates the Beclin-1/Bcl-2 complex (Bohensky et al., 2007), activates AMPK, inhibits mTOR (Bohensky et al., 2010), induces chondrocyte autophagy, and prevents apoptosis. P2X7R is also closely related to HIF-1α and autophagy (Mascanfroni et al., 2015). The ATP-P2X7R signal axis driven by oxidative metabolism participates in the differentiation of bone marrow-derived macrophages into their M2 type (Barbera-Cremades et al., 2016). HIF-1α also plays a key role in this process. For example, HIF-1α promotes lactic acid-dependent M2 polarization in the tumor microenvironment (Colegio et al., 2014). Interestingly, P2X7R is a strong stimulator of aerobic glycolysis and lactic acid production (Amoroso et al., 2012). P2X7R receives ATP signals to stimulate the alkalinization of lysosomes (Guha et al., 2013), which leads to an increase in the lysosomal pH. The accumulated autophagosomes cannot be fused with lysosomes for degradation and release outside the cell, thereby reducing autophagy flux (Takenouchi et al., 2009a, b). P2X7R activation downregulates the expression of glutamate transporter and promotes neuro-autophagy, which increases excitatory amino acids (Zhang et al., 2008; Działo et al., 2013; Kulbe et al., 2014) caused by an abnormal stimulation of glutamate receptors, ultimately resulting in cognitive impairment (Sun et al., 2015). P2X7R activates PI3K/Akt/GSK3β/β-catenin and/or mTOR/HIF1α/VEGF pathways to promote the proliferation and metastasis of osteosarcoma and increase bone destruction (Zhang et al., 2019).

#### Significance of P2X7R in Autophagy

Whether P2X7R promotes or inhibits autophagy is still controversial (Sluyter, 2017). For instance, one study showed ATP-activated P2X7R to inhibit the PI3K/AKT pathway, activate the AMPK-PRAS40-mTOR pathway, promote autophagy, inhibit cell proliferation, and exert anti-tumor effects (Bian et al., 2013). In another study, LL-37 activated AMPK and PI3K through P2X7R-mediated Ca^2+^ influx, promoted autophagy, and aided in inducing resistence against *Mycobacterium tuberculosis* in macrophages (Rekha et al., 2015). In another case, P2X7R-mediated Ca^2+^ influx activated mTOR and inhibited Treg cell conversion (Bohensky et al., 2010). However, to the contrary, in memory CD8+ T cells, P2X7R receives extracellular ATP signals to activate AMPK through Ca^2+^ influx and increase the AMP/ATP ratio, inhibit mTOR, and enhance mitochondrial function (Borges da Silva et al., 2018).

The level of autophagy is also related to the activation state of P2X7R. In the early stage of activation, P2X7R-mediated K^+^ efflux and Ca^2+^ influx activate mtROS, and Ca^2+^ activates CaMK, which in turn activates the AMPK pathway, inhibits mTOR, and promotes mitophagy and lysosome biogenesis. In the late stage of activation, lysosome stability decreases and cell death occurs (Sekar et al., 2018). Energy receptor AMPK (Garcia and Shaw, 2017) is also regulated by exercise (Garber, 2012). High-intensity exercise activates AMPKα to increase autophagic flux (Schwalm et al., 2015). In skeletal muscle, exercise induces mitophagy to degrade damaged mitochondria through the AMPK-Ulk1 signaling pathway (Laker et al., 2017). When energy is severely lacking, exercise inhibits AMPK/Ulk1/Beclin-1 phosphorylation, and the accumulated p62/SQATM1 inhibits autophagy to reduce muscle loss (Martin-Rincon et al., 2019). As an important downstream signaling molecule of P2X7R, AMPK is involved in the regulation of several physiological and pathological functions (Jeon, 2016), including autophagy in OA.

## Autophagy Is a Double-Edged Sword in OA

Compared with microautophagy and chaperone-mediated autophagy, macroautophagy is most studied and understood (Parzych and Klionsky, 2014). However, the relationship between cartilage damage, the degree of autophagy, and cell death remains unclear (Caramés et al., 2015).

### The Positive Side of Autophagy

The consensus is that autophagy exerts a protective effect on chondrocytes in OA. In mice, as cartilage damage increases, and resulting cell death ensues, the expression levels of autophagy-related genes decrease (Caramés et al., 2015). In cell lines and primary chondrocytes, decreased autophagy leads to cartilage degradation (Ribeiro et al., 2016a). Rapamycin (an autophagy inducer) promotes the degradation and clearance of damaged mitochondria, reduces IL-1β-induced ROS generation, and reduces the OA-like phenotype of chondrocytes (Sasaki et al., 2012). Decreased levels of autophagy are often accompanied by increased levels of apoptosis [e.g., presentation of activated PARP (the caspase-3 substrate)], further exacerbating OA characteristics (Caramés et al., 2015). Moreover, Beclin-1 silencing which reduces autophagy exacerbates cell death (Bohensky et al., 2007). mTOR overexpression also inhibits chondrocyte autophagy and promotes apoptosis, leading to increased cartilage degeneration (Zhang Y. et al., 2015). Autophagy promoted through oligomycin stimulation can effectively eliminate dysfunctional mitochondria, thereby protecting cells from apoptosis (Lopez de Figueroa et al., 2015).

Exercise promotes autophagy which relieves inflammation (Deretic et al., 2013). During exercise, the innate immune molecule TLR-9 interacts with Beclin-1 to strengthen and regulate AMPK activation in muscles (Liu Y. et al., 2020). AMPK interacts with sestrins to participate in exercise-induced autophagy to maintain skeletal muscle glucose metabolism (Liu et al., 2015), and relieve aging-related muscle atrophy (Fan et al., 2017). Exercise-induced AMPK activation can also inhibit mTOR, thereby alleviating other diseases as well by promoting autophagy, reducing the transformation of fatty liver to hepatitis and tumors (Guarino et al., 2020), and alleviating heart damage caused by exhaustive exercise (Liu and Pan, 2019).

### The Negative Side of Autophagy

Excessive autophagy can be a double-edged sword (Carames et al., 2010; Lopez de Figueroa et al., 2015; Ribeiro et al., 2016a, b), with no protective effect on cells (Chang et al., 2013), resulting in cell death (Shapiro et al., 2014) through synergistic participation in the process of cell apoptosis (e.g., ATP-dependent apoptosis). Related genes, such as *Ulk1*, *Beclin-1*, and *LC3*, are highly expressed in the early stage of OA, reflecting the protective effect of autophagy on chondrocytes; while in the late stage of OA, they are weakly expressed, as a consequence of autophagy-induced apoptotic cell death (Almonte-Becerril et al., 2010; Carames et al., 2010; Sasaki et al., 2012; Caramés et al., 2015). The opening of membrane pores induced by the ATP-P2X7R axis in muscle injury mediates autophagic cell death (Young et al., 2015). P2X7R receives ATP signals induced by ivermectin to promote autophagy, leading to tumor cell death. Although the role of P2X7R in infection and inflammation has been confirmed (Di Virgilio et al., 2017), studies have shown that inflammation leads to the downregulation of P2X7R expression, which in turn inhibits the PI3K-AKT-mTOR pathway, and promotes the osteogenesis of periodontal ligament stem cells (Xu et al., 2019).

### The Consensual Role of Autophagy in Pyroptosis

Contrary to the uncertain relationship between autophagy and apoptosis, autophagy usually alleviates pyroptosis (Gudipaty et al., 2018). This can be verified through various mechanisms in many studies. First, miR-103 targets BNIP3 (Bcl2/Adenovirus EIB 19 kDa Interacting Protein 3) to mediate late autophagy and relieve H_2_O_2_-induced oxidative stress and pyroptosis (Wang et al., 2020). Second, electrical stimulation affects THP-1 macrophages, activates Sirt3, promotes autophagy, and relieves ROS-induced pyroptosis (Cong et al., 2020). Third, adrenomedullin promotes autophagy and inhibits pyroptosis in testicular stromal cells through the ROS-AMPK-mTOR pathway (Li et al., 2019). Fourth, resveratrol reduces mitochondrial damage and increases autophagy, thereby inhibiting NLRP3 activation and reducing inflammation (Chang et al., 2015). Fifth, baicalein, a Chinese herbal ingredient, promotes autophagy degradation, and reduces unfolded protein accumulation and mitochondrial dysfunction caused by spinal cord ischemia-reperfusion injury, thereby alleviating pyroptosis (Wu et al., 2020). Sixth, metformin activates AMPK, inhibits mTOR, relieves pyroptosis, and treats diabetic heart disease in obese mice (Yang et al., 2019). And seventh, SP1 transcription increases the expression of lnc ZFS1, which downregulates miR-590-3p, inhibits AMPK, activates mTOR, inhibits autophagy, increases pyroptosis, and aggravates sepsis-induced cardiac dysfunction (Liu Y. et al., 2020).

In addition to removing harmful components in the cell, autophagy can also prevent pyroptosis by degrading inflammasome components. The NLRP3 inhibitor CP-456773 and the NF-κB inhibitor celastrol work together to induce autophagy through the AMPK-mTOR pathway and inhibit HSP-90, thereby increasing the autophagic degradation of NLRP3 to inhibit pyroptosis (Saber et al., 2020). Furthermore, bone marrow-derived mesenchymal stem cell (BMSC)-derived exosomes activate AMPK in hypoglycemic/reoxygenation-induced pheochromocytoma cells, inhibiting mTOR, promoting autophagic flux, while LC3 results in NLRP3 degradation to inhibit pyroptosis (Zeng et al., 2020). Also, immunity-related GTPase M (IRGM) interacts with NLRP3 and ASC to inhibit inflammasome oligomerization, thereby inhibiting its assembly and activation, and selectively degrades NLRP3 and ASC by autophagy, alleviating inflammatory cell death (Mehto et al., 2019).

Sometimes, autophagy is also the trigger point of pyroptosis. Autophagy mediates the release of IL-1β (Claude-Taupin et al., 2018). In macrophages, IL-1β is released outside the cell through a hole in the plasma membrane of N-GSDMD, while in neutrophils, N-GSDMD is not localized on the plasma membrane. IL-1β is released through the LC3^+^ autophagosome pathway (Karmakar et al., 2020). Arsenic can also promote autophagy, with lysosome degradation releasing cathepsin, resulting in NLRP3 activation (Qiu et al., 2018). Autophagy is often inhibited when pyroptosis occurs. During the resting state of macrophages, NLRC4 and Beclin-1/Atg6 form a complex to inhibit autophagy. Under a low degree of infection, NAIP5 recruits NLRC4 and pro-caspase-1 to form a complex to relieve autophagy inhibition, and induce cell protection. When autophagy cannot eliminate an intracellular infection, caspase-1 is activated to initiate pyroptosis (Byrne et al., 2013). NLRP3 inflammasomes in hepatocellular carcinoma cells inhibit autophagy through the 17β-estradiol (E2)/ ERβ/AMPK/mTOR pathway (Wei et al., 2019).

### Autophagy Discordance With Pyroptosis

When the two are activated together, autophagy negatively regulates pyroptosis. Inflammasome activation can induce autophagy, but the recruitment of LC3 and p62 induces the co-localization of inflammasomes with autophagosomes, thereby degrading them (Shi et al., 2012). Furthermore, when acrolein induces NLRP3 activation and autophagy through ROS, autophagy inhibits pyrolysis and mitochondrial damage (Jiang et al., 2018). Moreover, H_2_O_2_ induces nucleus pulposus cells to produce ROS, inducing autophagy, pyroptosis, and the upregulation of nuclear factor erythroid 2 like 2 (NFE2L2, Nrf2). Both Nrf2 and autophagy can alleviate pyroptosis and intervertebral disc degeneration (Bai et al., 2020). Tumor necrosis factor receptor-associated factor 3 (TRAF3) mediates the ubiquitination and degradation of Ulk1 and induces ROS and pyroptosis, while Ulk1 inhibits ROS and apoptosis inducing factor (AIF) translocation into the nucleus (Shen et al., 2020). In another mechanism, zearalenone inhibits the Akt/mTOR pathway to promote autophagy, while also promoting pyroptosis through NF-κB, but the upregulation of autophagy inhibits pyroptosis (Wang et al., 2019). In macrophages, NF-κB mediates the delayed accumulation of p62, forming the NF-κB-p62-mitophagy regulatory loop eliminating damaged mitochondria caused by pyroptosis, and limiting the intracellular pro-inflammatory activity (Zhong et al., 2016).

### Alternative Autophagy Mechanisms in Pyroptosis

Autophagolysosomes formed by the fusion of autophagosomes and lysosomes play an important role in pyroptosis. Lysosomes act as AMPK-mTOR signaling hubs and an instability may lead to apoptosis and pyroptosis (Zhu et al., 2020). An impaired autophagy-lysosomal pathway in macular corneal dystrophic cornea can also lead to pyroptosis (Zheng et al., 2020). Moreover, BpV(phen) increases the ubiquitination of p62, affects the binding of p62 and HDAC6, activates the deacetylation of α-tubulin, and affects the stability of acetylated microtubules, resulting in hindered autophagosome and lysosome fusion, while autophagy inhibition leads to apoptosis and pyroptosis (Chen et al., 2015). Lastly, hypoxia-induced autophagy/lysosomal dysfunction leads to ERS, leading to unfolded protein accumulation and impaired autophagy, which in turn activates NLRP3 inflammasomes and induces pyroptosis (Cheng et al., 2019).

### Significance of P2X7R in Cell Death Interplay

The interrelationships among phenotypes, such as autophagy, apoptosis, and pyroptosis, are also reflected in P2X7R-mediated cell metabolism and nutrition (Orioli et al., 2017). P2X4/P2X7/pannexin-1 mediates ROS, Ca^2+^/CaMK II, mitochondrial membrane potential, and caspase-1 activation caused by NADPH oxidase to promote cell pyroptosis and necrosis (Draganov et al., 2015). In ischemic stroke disease, P2X7R-mediated Ca^2+^ and K^+^ flow leads to mitochondrial dysfunction, caspase-8 and MAPK activation, and induces apoptosis. Ca^2+^ influx induces lysosomal dysfunction, which leads to decreased autophagic flow and apoptosis. K^+^ efflux leads to the activation of inflammasomes and induces pyroptosis (Zhao et al., 2018). Ca^2+^ overload, caused by P2X7R-mediated Ca^2+^ influx under the stimulation by high ATP concentrations in macrophages, leads to mitochondrial dysfunction, which in turn causes cell pyroptosis. However, when P2X7R is stimulated by low ATP concentrations or is positively allosterically regulated by compound K, the accumulation of mtROS and the activation of caspase-1 and 3 alter cell death mechanism from pyroptosis to apoptosis (Bidula et al., 2019).

In summary, autophagy primarily plays the role of a protector, resisting stimuli that cause damage to cells through key signaling pathways, such as AMPK-mTOR and HIF-1α, and supporting homeostasis. When autophagy decreases, P2X7R-mediated ion flux and damage to organelles, such as mitochondria and lysosomes, prevent autophagy flux, and the balance shifts. At this time, autophagy may induce or convert into apoptosis and pyroptosis. On several occasions, autophagy is the initiator of apoptosis, pyroptosis, and inflammation. Therefore, autophagy is a double-edged sword, and the right balance, together with proper activation of P2X7R, is the key to its power.

## Therapeutic Significance of P2X7R Inhibitors

The P2X7 receptor inhibition can occur via synthetic reagents, ions, natural molecular compounds, and Chinese herbal medicines. However, due to P2X7R’s central role in inflammation, P2X7R inhibitors are receiving more attention for the development of targeted receptor therapies (Khakh and North, 2006; Arulkumaran et al., 2011; North and Jarvis, 2013; Bartlett et al., 2014; Di Virgilio et al., 2017). Various inhibitors display differences in chemical structure, species selectivity, competitive or non-competitive antagonistic methods, and specificity. The first-generation inhibitors, such as Reactive Blue 2, KN-62, PPADS, brilliant blue G (BBG), and oxidized ATP (oATP), are not highly selective, inhibiting other purinergic receptors as well. Second-generation inhibitors have improved target specificity for P2X7R, such as A438079, A740003, A839977, AZ10606120, AZ11645373, GSK314181, and JNJ-47965567. To date, most *in vivo* experiments have been conducted with first-generation inhibitors, but a select few studies have been carried out with specific inhibitors, like A438079, showing good efficacy *in vitro* and *in vivo*. Pretreatment of a DC/CD4+ T cell co-culture system with A438079, and the ensuing inhibition of P2X7R, can reduce the levels of pro-inflammatory factors IL-1β, IL-6, IL-23p19, and TGF-β1, derived from Th17 cells. In the arthritis mice model, induced by the activation of related collagen, A438079 relieved the swelling of the hind paw and the pathological changes in the ankle joint (Fan et al., 2016).

In view of the positive therapeutic effects of P2X7R inhibitors in rodent studies, drugs targeting human P2X7R are being used in clinical settings for the treatment of several diseases, such as pain, arthritis, and multiple sclerosis (Bartlett et al., 2014). The therapeutic effects of inhibitors, such as oATP, BBG, KN-62, and A438079, have been confirmed in preclinical models of inflammatory diseases, such as contact allergy, inflammatory pain, endotoxin-induced fever, and antibody-induced nephritis (Matute et al., 2007; Taylor et al., 2009; Weber et al., 2010; Barbera-Cremades et al., 2012). Two clinical trials of patients with rheumatoid arthritis receiving P2X7R-specific inhibitors, AZD9056 and CE-224, reported on its safety and clinical efficacy. The dosages were well tolerated, but efficacy was not improved in patients already resistant to methotrexate or sulfasalazine (used in the treatment of joint swelling and pain) (Keystone et al., 2012; Stock et al., 2012). This would point to a need to use P2X7R inhibitors in conjunction with other targeted therapies capable of resensitizing mechanistic pathways involved in drug-resistance.

Although rodent models and receptor inhibitors are regularly encountered in P2X7R research, the selectivity and specificity of inhibitors still require attention. Recent development of therapeutic antibodies, such as nanobodies or single-domain antibodies, can also have the potential to specifically inhibit membrane proteins. Therefore, we expect the development of more effective drugs and treatments targeting P2X7R.

## Conclusion and Prospects

**FIGURE 1:**
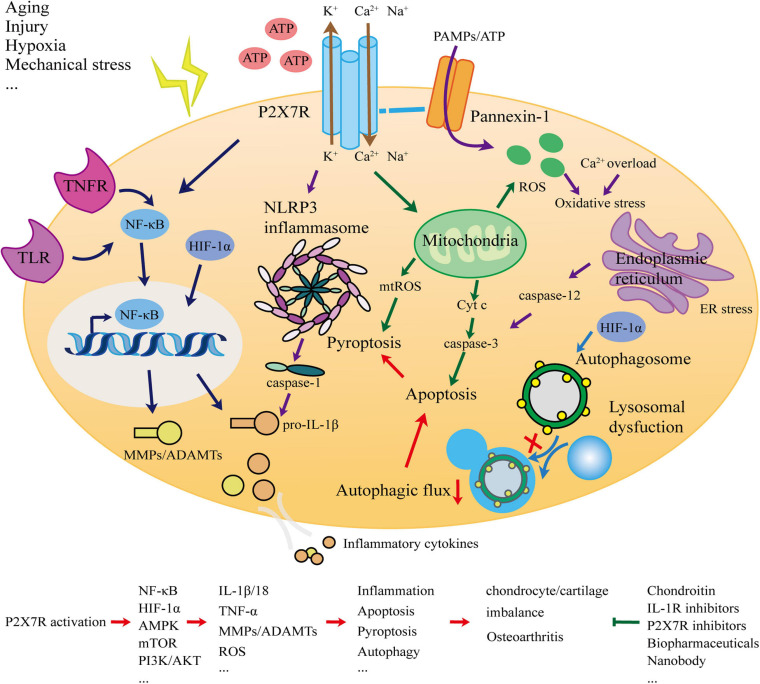
Schematic for the role of the P2X7 receptor in osteoarthritis.

This article systematically analyzed and elucidated the association between P2X7R and OA. The most typical manifestation of OA is cartilage degradation. As the only cellular component of cartilage, chondrocytes are in a stable state and balance is crucial. The ECM degradation of chondrocytes and the release of inflammatory factors are important events leading to cartilage degradation. Inflammation is a direct manifestation of OA. To explore whether P2X7, as a key switch of inflammation, is involved in the occurrence and development of OA, we considered the network (Figure 1) of interaction from the perspectives of inflammation, apoptosis, pyroptosis, and autophagy, and presented new OA prevention and treatment strategies.

Currently, there is no treatment method that can completely prevent the occurrence and development of OA. Delaying and reducing the death of chondrocytes to prevent the degradation of the cartilage matrix could be a potential therapeutic focus. As various factors affect chondrocyte cell death progression, including the degree of cartilage degradation, pro-inflammatory cytokines, and ATP availability, we propose the therapeutic focus to begin with the recognition of ATP as the first step in activating inflammation to narrow our approach to anti-inflammatory designs. Caspase-knockout studies have found that cells require both ATP energy and caspases to transform pyroptosis into apoptosis, thereby moving toward programmed death, and away from necrosis (Zeiss, 2003). zVAD-fmk is often used as a caspase inhibitor in OA research (Hwang and Kim, 2015), which can significantly reduce chondrocyte apoptosis and cartilage degradation (D’Lima et al., 2006). Moreover, when these factors are managed, mitochondrial integrity remains intact, reducing oxidative stress levels. Retaining a positive antioxidant balance exerts anti-inflammatory and anti-apoptotic effects. Oxidative stress can further be balanced by promoting autophagy, thus promoting mitochondrial housekeeping. However, autophagy can be a double-edged sword, and needs careful consideration when promoted. Excessive autophagy can trigger cell apoptosis, and the rapid loss of chondrocytes may worsen OA (Shapiro et al., 2014). Nevertheless, the mainstream view remains that autophagy stimulation in the early stages of OA can protect chondrocytes.

Treatment strategies should also focus on the role of P2X7R. Soon after it was cloned, the P2X7R protein received widespread attention as a key switch for inflammation with great therapeutic potential. For some chronic inflammatory diseases, small molecule (drug-like) inhibitors targeting P2X7R have been used in phase I and II clinical studies (Gunosewoyo and Kassiou, 2010; Sluyter and Stokes, 2011; Park and Kim, 2017). To date, more than 30 clinical studies in this regard have been conducted. Although the safety of inhibitors has been satisfactory, the clinical efficacy has been disappointing. In knee OA, rheumatoid arthritis, and chronic obstructive pulmonary disease, the efficacy is poor, while in Crohn’s disease fairly positive (Arulkumaran et al., 2011; Keystone et al., 2012; Stock et al., 2012; Eser et al., 2015). Biopharmaceuticals may present a new way to replace small drug-like compounds. Antibodies against non-functional variants of human P2X7R have been used to treat cancer. The highly purified goat IgG can effectively reduce pathological changes in basal cell carcinoma size (Gilbert et al., 2017). In addition, nanobodies (e.g., 13A7 nanobody, single-domain antibody fragments elicited in camelids) that can bind to human or mouse P2X7R with high affinity, after inoculation in mice, effectively relieve the symptoms of experimental allergic contact dermatitis and glomerulonephritis. Dano1 nano antibody is selective for human P2X7R and can effectively reduce the level of IL-1β in the blood after endotoxin treatment (Danquah et al., 2016). Therefore, in the case of poor efficacy of anti-P2X7 receptor active drugs, biologics targeting P2X7R indicate a new direction for future research.

In conclusion, there remains a need for more specific OA therapies to be developed. P2X7R of the purinoceptor family, is closely related to inflammation and a promising drug target for OA. However, research into P2X7R and its role in the pathogenesis of OA requires further investigation. For the benefit of patients and scientific progress, we believe that these expectations will be realized in the near future.

## Author Contributions

ZL performed the literature review, drafted the manuscript, and prepared the figure. ZH and LB edited and revised the manuscript. All authors contributed to the article and approved the submitted version.

## Conflict of Interest

The authors declare that the research was conducted in the absence of any commercial or financial relationships that could be construed as a potential conflict of interest.
